# Condylar positioning changes following unilateral sagittal split ramus osteotomy in patients with mandibular prognathism

**DOI:** 10.1186/s40902-015-0036-y

**Published:** 2015-10-16

**Authors:** Myung-In Kim, Jun-Hwa Kim, Seunggon Jung, Hong-Ju Park, Hee-Kyun Oh, Sun-Youl Ryu, Min-Suk Kook

**Affiliations:** Department of Oral and Maxillofacial Surgery, School of Dentistry, Dental Science Research Institute, Chonnam National University, 77, Yongbongro, Buk-Gu, Gwangju 500-757 South Korea

## Abstract

**Background:**

This study was performed to evaluate three-dimensional positional change of the condyle using three-dimensional computed tomography (3D-CT) following unilateral sagittal split ramus osteotomy (USSRO) in patients with mandibular prognathism.

**Methods:**

This study examined two patients exhibiting skeletal class III malocclusion with facial asymmetry who underwent USSRO for a mandibular setback. 3D-CT was performed before surgery, immediately after surgery, and 6 months postoperatively.

After creating 3D-CT images by using the In-vivo 5™ program, the axial plane, coronal plane, and sagittal plane were configured. Three-dimensional positional changes from each plane to the condyle, axial condylar head axis angle (AHA), axial condylar head position (AHP), frontal condylar head axis angle (FHA), frontal condylar head position (FHP), sagittal condylar head axis angle (SHA), and sagittal condylar head position (SHP) of the two patients were measured before surgery, immediately after surgery, and 6 months postoperatively.

**Results:**

In the first patient, medial rotation of the operated condyle in AHA and anterior rotation in SHA were observed. There were no significant changes after surgery in AHP, FHP, and SHP after surgery. In the second patient, medial rotation of the operated condyle in AHA and lateral rotation of the operated condyle in FHA were observed. There were no significant changes in AHP, FHP, and SHP postoperatively. This indicates that in USSRO, postoperative movement of the condylar head is insignificant; however, medial rotation of the condylar head is possible. Although three-dimensional changes were observed, these were not clinically significant.

**Conclusions:**

The results of this study suggest that although three-dimensional changes in condylar head position are observed in patients post SSRO, there are no significant changes that would clinically affect the patient.

## Background

Orthognathic surgery has become increasingly popular in recent years with the development of general anesthesia using nasotracheal intubation, improvement of miniplates, fixation screws, and surgical instruments, reduction of operating time, and the increased efforts to minimize complications and side effects, regardless of expenses [1].

The first commonly known surgical correction of jaw deformities was carried out by Hullihen in 1849 in the USA and the correction of mandibular prognathism by using body osteotomy to allow retrusion of the mandible was first reported by Blair in 1906 [2, 3]. Limberg described subcondylar osteotomy using an intraoral approach, and Obwegeser and Trauner popularized sagittal split ramus osteotomy (SSRO) through systematic development and modification of the technique [4, 5]. Later, Dal Pont, Hunsuck, Bell et al., and Epker introduced modified techniques that popularized SSRO along with the intraoral vertical ramus osteotomy (IVRO), and the SSRO went on to become one of the most commonly used surgical techniques [6–9].

SSRO can easily relocate the distal fragment, including the teeth, and the large contact surface of the relocated mandible makes it easy for the bones to connect and recover which results in minimal positional changes of the temporomandibular joint and masticatory muscles. However, some disadvantages of SSRO include positional changes of the condylar head, relapse, malocclusion, unintentional fractures, and neurovascular injury. Postoperative positional changes of the condylar head, in particular, increase the risk of temporomandibular dysfunction, malocclusion, relapse, etc.

Bilateral sagittal split ramus osteotomy (BSSRO) has the same disadvantages while unilateral sagittal split ramus osteotomy (USSRO) has the advantage of being less invasive and having fewer complications. However, after this type of surgery, there are more chances of the condylar position changing, leading to higher risk of temporomandibular disorder, from a non-operative aspect.

Harris et al. observed that the condylar head had a tendency to move in a medio-posterior-superior direction and rotate inwardly post SSRO with rigid fixation (RF) for correction of mandibular retrognathism [10]. They also reported that the condylar displacement was related to the amount of mandibular protrusion, the rotation of the proximal bone segment, and the form of the mandible. However, Hackney et al., in their study examining differences in the magnitude of advancement, temporomandibular symptoms, and the shape of the mandible in patients with mandibular retrognathism, found no significant correlation between the amount of mandibular movement and postoperative condylar position [11].

In the past, there have been difficulties in conducting three-dimensional (3D) reconstructions using computed tomography (CT). The recent CT scan and 3D programs, however, help reduce errors created by the patient’s movement, allow accurate measurements by minimizing errors caused by magnification or distortion of the image, limit images to rotating at a specific site and along a manipulating axis, and make it possible to observe deeper structures by excluding surface structures when needed, thus offering more benefits than two-dimensional measurements. Many studies have used CT images to examine positional changes of the condylar head after BSSRO operations. However, there are very few that examine positional changes of the condylar head after USSRO. Prospective research should be designed to reveal the high prevalence of positional changes in non-operated condylar heads, associated with increased risk of temporomandibular dysfunction post USSRO operation.

The purpose of this study was to evaluate three-dimensional positional changes of the condyle using 3D-CT after USSRO.

## Case presentation

### Methods

This study was performed in accordance with the Institutional Guidelines of Chonnam National University Dental Hospital (CNUDH-EXP-2014-007).

To evaluate the condylar positional changes after unilateral sagittal split ramus osteotomy, CT scans were taken preoperatively and postoperatively. These scans were then reconstructed into three-dimensional images and evaluated using the In-vivo 5™ program. The variables were measured using CT scans. To minimize errors, measurements were taken three times from the same person, and the average values were calculated.

**Fig. 1:**
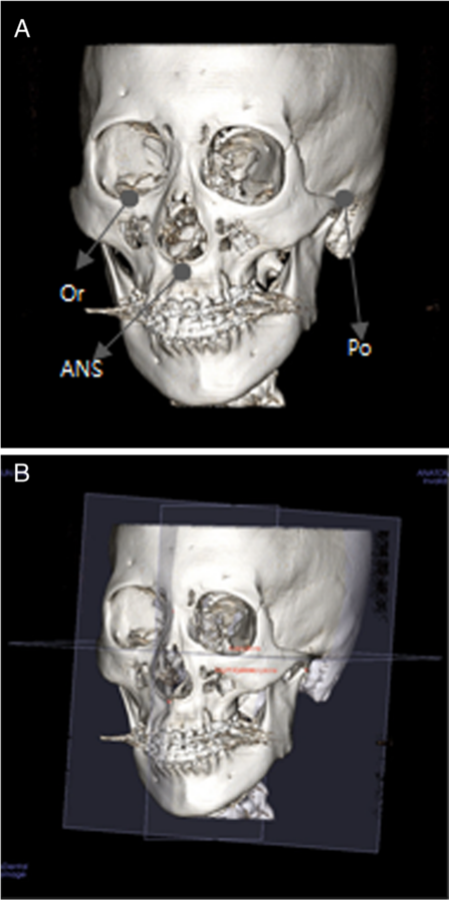
Anatomical landmarks and reference planes in 3D-CT: **a** reference points and **b** reference planes

**Fig. 2:**
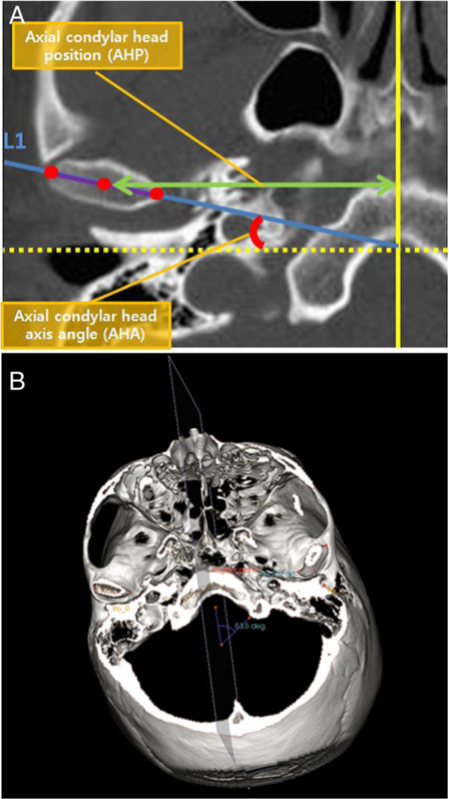
In the axial view, the slice that showed the greatest mediolateral dimension of the condylar head was selected. Axial condylar head long-axis line was drawn along the axis of the condylar head from the lateral pole to the medial pole (**a**, **b**)
- Axial condylar head position (AHP): perpendicular distance between the sagittal plane and the most medial point of the condylar head (Fig. 2)
- Frontal condylar head long-axis angle (FHA): angle between the axial plane and the frontal condylar head long-axis line which connects the medial and lateral poles of condylar head (Fig. 3)

1. Computed tomography and In-vivo 5™ program measurements
  Computed tomography (CTI Pro, GE Co., USA) of the facial bone was carried out, and the CT scans were converted and saved as Digital Imaging and Communications in Medicine (DICOM) files. These were then reconstructed using the In-vivo 5™ program (Fig. 1). Reconstructed three-dimensional images of the axial plane, coronal plane, and sagittal plane were then configured to observe changes in the condylar position and angle.

2. Reference points (Fig. 1a)

- Orbitale (Or): mid-point of the infraorbital margin
- Anterior nasal spine (ANS): tip of the anterior nasal spine
- Porion (Po): superior point of the external auditory meatus

3. Reference planes (Fig. 1b)

- Axial plane: plane containing both porions and the right orbitale
- Coronal plane: plane perpendicular to axial plane that includes both porions
- Sagittal plane: plane perpendicular to axial and coronal planes that includes ANS

4. Measurement data

- Axial condylar head long-axis angle (AHA): angle between the sagittal plane and the axial condylar head axis line which connects the medial and lateral poles of the condylar head (Fig. 2)

**Fig. 3:**
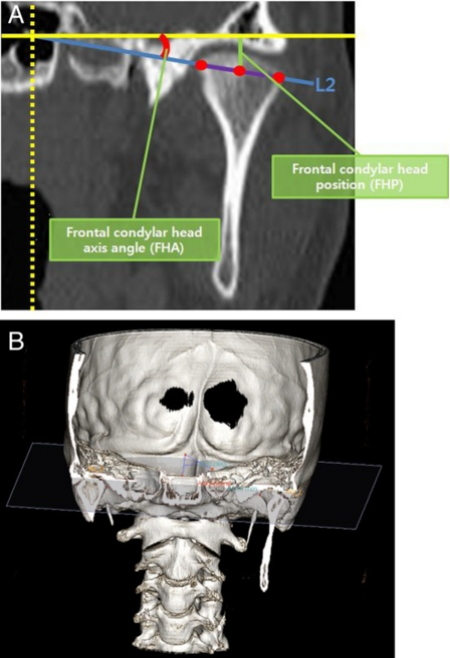
In the frontal view, the slice that showed the greatest mediolateral dimension of the condylar head was selected. Frontal condylar head long-axis line was drawn along the axis of the condylar head from the lateral pole to the medial pole (**a**, **b**)
- Frontal condylar head position (FHP): perpendicular distance between the axial plane and most superior point of the condylar head (Fig. 3)
- Sagittal condylar head long-axis angle (SHA): angle between the coronal plane and the sagittal condylar head long-axis line which connects the most posterior point of condylar head and the point at the level of the sigmoid notch (Fig. 4)

**Fig. 4:**
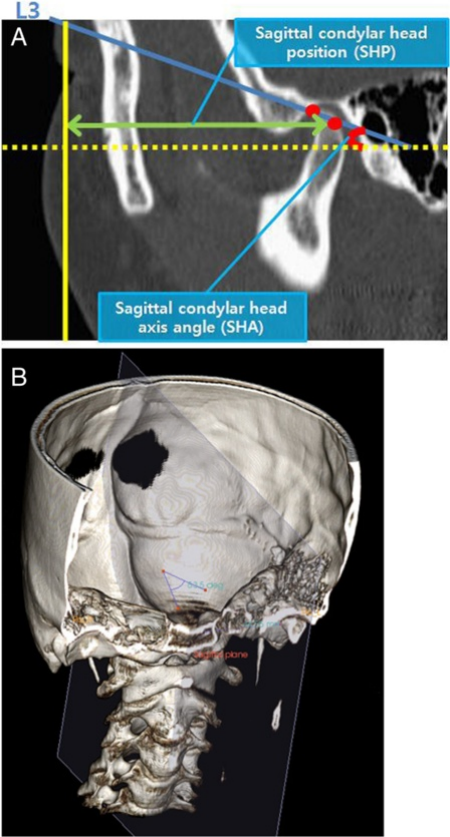
In the sagittal view, the slice that showed the greatest anteroposterior dimension of the condylar head was selected. Sagittal condylar head long-axis line, parallel to condyle neck inclination was drawn from the most superior point of condyle head (**a**, **b**)
- Sagittal condylar head position (SHP): perpendicular distance between the coronal plane and the most superior point of the condylar head (Fig. 4)

### Results

#### Case 1

**Fig. 5:**
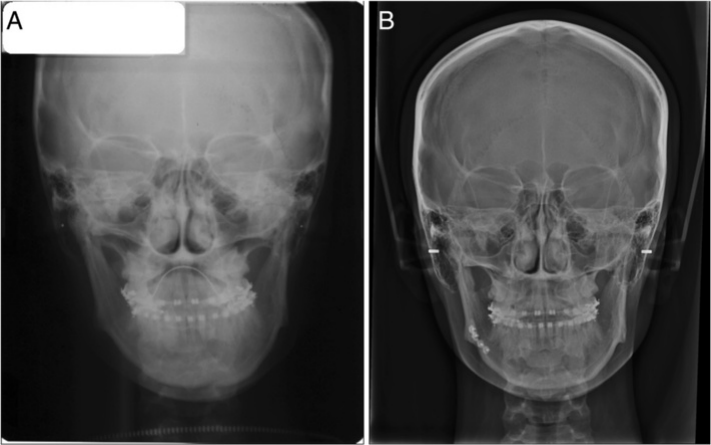
Radiographic findings of case 1: **a** pre-operation and **b** post-operation

A 20-year-old female patient with skeletal class III malocclusion but without any TMJ symptoms underwent USSRO under general anesthesia at the Department of Oral and Maxillofacial Surgery, Chonnam National University Hospital. There was a setback of 6 mm to the right, with no additional surgery (Fig. 5). No sounds in the temporomandibular joint, pain, or limited opening was detected after surgery. CT scans were taken preoperatively, immediately after the operation, and 6 months postoperatively and evaluated (Tables 1 and 2).

**Table 1:** Positional change of the condyle in case 1

Variables		AHP	FHP	SHP
Osteotomy	Pre-op	41.6 ± 0.2	4.2 ± 0.5	12.1 ± 0.9
	Post-op	41.6 ± 1.0	3.8 ± 0.2	11.8 ± 0.8
	Post-op 6M	42.4 ± 0.3	2.9 ± 0.5	11.8 ± 0.6
Non-osteotomy	Pre-op	43.0 ± 0.2	3.9 ± 0.5	10.8 ± 0.3
	Post-op	42.8 ± 0.9	2.6 ± 0.6	9.4 ± 1.3
	Post-op 6M	41.4 ± 0.2	3.4 ± 0.4	10.4 ± 0.6

*AHP* axial condylar head position, *FHP* frontal condylar head position, *SHP* sagittal condylar head position, *Pre*-*op* before operation, *Post*-*op* after operation, *Post*-*op 6M* 6 months after operation

**Table 2:** Angular change of the condyle in case 1

Variables		AHA	FHA	SHA
Osteotomy	Pre-op	66.3 ± 1.0	17.0 ± 0.5	10.7 ± 0.8
	Post-op	63.3 ± 0.9	17.8 ± 0.6	11.0 ± 1.3
	Post-op 6M	62.5 ± 1.5	21.2 ± 1.0	10.7 ± 2.5
Non-osteotomy	Pre-op	59.5 ± 0.5	21.3 ± 0.6	10.1 ± 1.5
	Post-op	55.3 ± 1.3	22.9 ± 1.9	15.1 ± 2.6
	Post-op 6M	59.2 ± 1.8	21.2 ± 1.9	11.0 ± 1.1

*AHA* axial condylar head axis angle, *FHA* frontal condylar head axis angle, *SHA* sagittal condylar head axis angle, *Pre*-*op* Before operation, *Post*-*op* After operation, *Post*-*op 6M* 6 months after operation

The amount of change in AHA, FHA, and SHA indicated that there was a lateral rotation in axial, frontal, and sagittal aspects of the condylar heads in the surgical region only. However, there was no significant positional change in either side.

#### Case 2

**Fig. 6:**
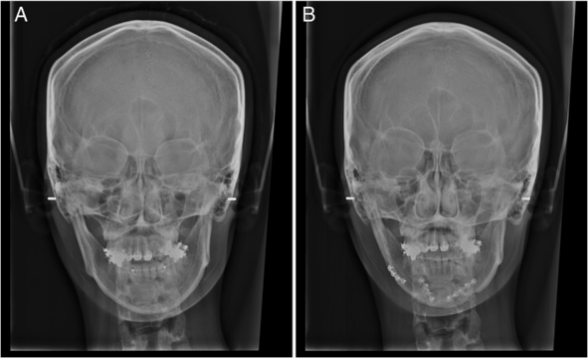
Radiographic findings of case 2: **a** pre-operation and **b** post-operation

An 18-year-old female patient with skeletal class III malocclusion underwent USSRO with a 4-mm setback at the right surgical site. Additionally, genioplasty, reduction malarplasty, and mandibular contouring surgery were performed (Fig. 6). Postoperatively, no sounds in the temporomandibular joint, pain, or limited opening was detected. CT scans were taken preoperatively, immediately after the operation, and 6 months postoperatively and evaluated (Tables 3 and 4).

**Table 3:** Positional change of the condyle in case 2

		AHP	FHP	SHP
Osteotomy	Pre-op	42.1 ± 0.4	1.9 ± 0.3	9.9 ± 0.3
	Post-op	42.8 ± 1.2	1.8 ± 0.4	9.0 ± 0.4
	Post-op 6M	41.9 ± 0.3	1.7 ± 0.9	10.4 ± 1.9
Non-osteotomy	Pre-op	43.6 ± 0.5	1.6 ± 0.5	8.4 ± 1.9
	Post-op	43.8 ± 1.7	1.5 ± 3.0	8.7 ± 1.7
	Post-op 6M	42.7 ± 0.5	1.3 ± 0.3	7.6 ± 0.9

*AHP* axial condylar head position, *FHP* frontal condylar head position, *SHP* sagittal condylar head position, *Pre*-*op* before operation, *Post*-*op* after operation, *Post*-*op 6M* 6 months after operation

**Table 4:** Angular change of the condyle in case 2

Variables		AHA	FHA	SHA
Osteotomy	Pre-op	70.5 ± 3.5	11.4 ± 0.7	12.3 ± 1.7
	Post-op	71.5 ± 2.1	12.8 ± 3.3	10.3 ± 1.5
	Post-op 6M	64.9 ± 0.9	11.1 ± 1.0	13.7 ± 3.9
Non-osteotomy	Pre-op	52.9 ± 1.8	13.1 ± 1.0	16.3 ± 0.6
	Post-op	53.4 ± 2.3	13.7 ± 1.6	18.0 ± 2.0
	Post-op 6M	52.1 ± 0.4	13.9 ± 1.5	17.1 ± 1.9

*AHA* axial condylar head axis angle, *FHA* frontal condylar head axis angle, *SHA* sagittal condylar head axis angle, *Pre*-*op* Before operation, *Post*-*op* After operation, *Post*-*op 6M* 6 months after operation

The amount of change in AHA, FHA, and SHA indicated that lateral rotation was observed in the axial aspect of the condylar head of the nonsurgical region, frontal, and sagittal aspects of the condylar head in the surgical region. However, there was no significant positional change in either side.

### Discussion

Lee and Park introduced post-surgical occlusion and condylar positions as the crucial factors influencing relapse [12]. Occlusion is adjustable through orthodontic treatment after surgery. However, the condylar position cannot be adjusted after surgery, and changes in the condylar position could lead to distorted occlusion. Moreover, the surgeon’s level of experience, the direction of movement of the mandibular distal fragment, the anatomical form of the mandible, and the fixation method used are factors that influence the condylar position after surgery.

The post-surgical condylar position could also change due to the movement of the mandibular distal fragment in a forward or backward direction. According to Will et al., both condyles had a tendency to move superiorly in 41 patients who underwent mandibular advancement surgery using SSRO, and according to Freihofer and Petresevic, both condyles had a tendency to move anteriorly in 38 patients who underwent mandibular advancement surgery using SSRO [13, 14]. Harris et al. reported that when mandibular advancement surgery was performed, the condyles moved inferiorly, superiorly, and posteriorly, and rotated inferiorly, while Hu et al. reported that the condyles were displaced posteriorly and rotated anteriorly in 22 patients who underwent mandibular advancement surgery using SSRO [10, 15].

It was reported that methods of fixating bone fragments could also influence the condylar position. According to Kundert and Hadjianghelou, the condyle had a tendency to rotate and incline when rigid fixation was performed, as observed in 35 patients who underwent SSRO [16]. Kawamata et al. reported that when computed tomography was performed 3 to 6 months after rigid fixation on patients with mandibular prognathism, the major axis of the condylar neck had a tendency to incline backwards, and the major axis of the condylar head had a tendency to incline laterally [17]. Furthermore, the condylar position of the mandible was evaluated using model jawbones constructed from computed tomography scans taken before and after surgery, and it was reported that the condylar heads had moved backwards by 1~2 mm and the distance between them had increased by 2 mm.

Past studies have used 2D radiographic photos to analyze the condylar position; however, difficulties arose due to overlapping images of both condyles and the position of the image changing with the position of the patient. However, since the introduction of CT, condyles could be observed without overlap, and the position of the patient had become irrelevant. As a result, analytical errors have reduced over time and more accurate analyses have become possible.

In the study of mandibular condyle after SSRO using three-dimensional computed tomography, Lee and Park reported that the condyle had moved inferiorly, rotated medially from the axial view, and moved posteriorly from the sagittal view [12]. However, the amount of posterior movement and the change of position exhibited by the mandibular condyle have no statistical correlation. Baek et al. reported that even though the amount of mandibular posterior movement for each side is different, there is no three-dimensional change in the joint [18].

In this study, we evaluated the change in mandibular condylar position after the migration of the distal fragment in patients with skeletal class III malocclusion who had undergone orthodontic treatment prior to surgery, did not exhibit asymmetric maxilla, and were in need of rotation on the non-operated side only. This was carried out by measuring AHA, AHP, FHA, FHP, SHA, and SHP. The post-surgical observation period in this study was much longer than that in any of the previous studies.

Previous studies such as those by Lee and Park and Baek et al. used a reference line for measurement [12, 18]. This study differs from the previous ones by using a reference plane set on a three-dimensional image, which is considered to help reduce errors in measurement over time.

Various methods have been introduced to maintain the position of the mandibular condyle in SSRO. Loenard introduced a method using acrylic and wire on the arch bar of the maxilla [19]. A variety of methods were later introduced by Nickerson and Epker and Wylie [20, 21]. However, Will et al. and Jaager et al. reported that using these techniques to maintain the condylar position did not have any significant result [13, 22]. In order to minimize the change in condylar position during surgery, the following methods were used in this study: intermaxillary fixation was performed before the mediolateral bone fragment was separated, a hole was drilled on the anterior rim of the mandibular ascending ramus on each side, and the position of the mandibular condyle was marked with a condylar positioner, using an orthodontic bracket on the maxilla as a landmark. The interfering bone region of the mediodistal bone fragment was removed prior to fixing the distal bone fragment post migration, to minimize displacement.

In the first patient, medial rotation of the operated condyle was observed in AHA, and anterior rotation was observed in SHA. Medial rotation in AHA is in accordance with Lee and Park’s study [12]. Anterior rotation is in accordance with Hu et al.’s study, in which the condylar head showed anterior rotation, and with Kawamata et al.’s study, in which posterior tilting of the condylar cervical axis was observed [15, 17]. In the second patient, the operated condyle rotated medially in AHA, laterally in FHA, and did not show any significant change in SHA. Changes in FHA are similar to the results seen in Kawamata et al.’s study, in which the condylar head was tilted laterally [17]. In both cases of USSRO, the postoperative movement of the condylar head was not significant. In spite of the slight three-dimensional changes being observed, they were not clinically significant.

Since USSRO patients did not have discomfort in their TMJ, the procedure is thought to be safe. Moreover, it is a time-efficient procedure. Condylar head displacement is a drawback of this method; however, positional changes were insignificant in our study. Therefore, it can be considered as an efficient method for patients with facial asymmetry requiring 1 or 2 mm advancement or setback on one side.

This study has reported the changes in condylar head position preoperatively, immediately after surgery, and 6 months postoperatively, using three-dimensional computed tomography, thus, conducting a longer follow-up observation compared to those of previous studies. However, for more reliable results, it is recommended that additional evaluation of changes in the condylar position be carried out using a larger sample of patients and a longer follow-up period.

## Conclusions

The results of this study suggest that although three-dimensional changes in the condylar head are observed post USSRO, there are no significant changes that would clinically affect patients. Therefore, when used in patients with proper indications, USSRO can be clinically useful.

## Consent

This case report was reviewed by Institutional Review Board (IRB) of Chonnam National University Dental Hospital and was exempted from deliberation.

## Abbreviations

3D-CT: three-dimensional computed tomography
AHA: axial condylar head long-axis angle
AHP: axial condylar head position
ANS: anterior nasal spine
FHA: frontal condylar head long-axis angle
FHP: frontal condylar head position
Or: orbitale
Po: porion
SHA: sagittal condylar head long-axis angle
SHP: sagittal condylar head position
USSRO: unilateral sagittal split ramus osteotomy
